# Do adolescents with hearing loss use social media and the internet differently from their hearing peers?

**DOI:** 10.1017/S002221512400149X

**Published:** 2025-03

**Authors:** Deniz Tuz, Busra Altın, Merve Ozbal Batuk

**Affiliations:** 1 Department of Audiology, Faculty of Health Sciences, Ege University, İzmir, Turkey; 2 Department of Audiology, Faculty of Healthy Sciences, Hacettepe University, Ankara, Turkey

**Keywords:** internet use, internet, hearing loss, cochlear implants, hearing aids

## Abstract

**Objective:**

The aim of this study was to compare the internet and social media use of teenagers with hearing loss with that of their normal hearing peers.

**Methods:**

The study included 27 hearing-impaired and 27 normal-hearing peers (12–18 years). The Social Media Attitude Scale, the Internet Use Purposes Scale, University of California, Los Angeles Loneliness Scale and the Problematic Internet Use Scale were used to compare hearing-loss and normal-hearing groups.

**Results:**

The social isolation subscale and Social Media Attitude Scale total score differed between groups (*p* = 0.001 and *p* = 0.048, respectively). Internet Use Purposes Scale education subscale differences were statistically significant (*p* = 0.042). Negative consequences (*p* = 0.007), excessive use (*p* = 0.021) and Problematic Internet Use Scale total score (*p* = 0.005) differed significantly. The University of California, Los Angeles Loneliness Scale had a moderate negative connection with the Problematic Internet Use Scale's social benefit/comfort subscale and total score (*r* = 0.369, *p* = 0.006 and *r* = −0.309, *p* = 0.023, respectively).

**Conclusion:**

While adolescents with hearing loss have limited online educational resources, problematic internet use is a concern. When overused, the internet can reduce loneliness, but it can also have harmful consequences.

## Introduction

As an alternative to the physical world, where face-to-face communication is essential, the internet provides a platform for widespread participation in various activities, such as socialising, meeting, conversing, learning and shopping, in a more comfortable and secure environment. Social media, the most popular of these methods, commonly described as web-based technologies or software applications, allows for connection, communication and shared multimedia-based content through online communication channels.^1,2^

From a technological standpoint, social media allows users to create new profiles, make friends, create content and create pages and groups while remaining behind the keyboard. They do not need to engage in face-to-face communication.^3^ Indeed, people who are dissatisfied with their physical characteristics, such as height and weight, or who suffer from voice disorders, have stated in previous studies that they feel more comfortable, confident and ‘normal’ when communicating online.^4,5^ It is also known that people with hearing loss have a similar tendency.^6^

Even though the prevalence of hearing loss increases with age, it remains one of childhood's most important and frequently encountered problems.^7,8^ It is thought that globally 34 million children have hearing problems, affecting their quality of life.^9^

Generally, the effectiveness of interventions such as hearing aids and cochlear implants may change depending on the type and degree of hearing loss, the choice of intervention and the accuracy of the fitting strategies. While some interventions can help children to achieve the communication and academic skills of their peers with normal hearing, in some cases children do not use their devices effectively or hearing close to normal hearing cannot be achieved. Aside from these issues, the appearance of devices can cause embarrassment, depression, exclusion and social isolation in the individual.^10^

Hearing-impaired people can use the internet for various purposes, including communication, learning, teaching, education and participation in online psychotherapy support groups and sign language communication. They can socialise on platforms including email, forums and social media just like people with normal hearing, without specifying their health conditions.^4,6^ This is an important area of research because internet and social media use has become increasingly prevalent among adolescents, and it is important to understand how hearing loss may impact their use and behaviours online. This study aimed to provide valuable insights into potential differences or challenges that may arise by comparing the internet and social media use of adolescents with hearing loss to their peers with normal hearing.

This research aims to address the inconsistencies in existing literature regarding social media usage patterns among individuals with hearing loss compared with those with normal hearing. While some studies suggest similarities in social media involvement between deaf and hard of hearing people and their hearing counterparts,^2,11^ one study demonstrated differences impacted by the presence of deafness or degree of hearing loss.^12^ In light of these differences, our study aimed to compare the internet and social media use of adolescents with implants and hearing aids, as well as those who do not rely on sign language, to that of their normally hearing peers.

## Materials and methods

The Ethics Committee of Hacettepe University approved this study (GO 21/158). The study was conducted according to the Helsinki Declaration, and all participants and caregivers provided written consent forms.

### Participants

The participants in this study included 27 hearing-impaired and 27 normal-hearing peers between the ages of 12 and 18 years. The inclusion criteria for the study group were as follows: (1) having a diagnosis of mild to profound hearing loss and using a cochlear implant or hearing aid; (2) having received a hearing loss diagnosis before the age of 4 years; (3) using a hearing device regularly and benefiting from it; (4) having a pure tone average of 50 dB or better with a hearing device; (5) being a native Turkish speaker; and (6) not having any additional diagnosed disabilities.

The inclusion criteria for the control group were as follows: (1) having normal tympanometry findings; (2) having 20 dB hearing loss or better hearing sensitivity at audiometric test frequencies of 250, 500, 1000, 2000, 4000 and 6000 Hz, and speech discrimination scores above 90 per cent; (3) aged 12–18 years; (4) being a native Turkish speaker; and (5) not having any additional diagnosed disability.

### Study design

Individuals who met the inclusion criteria for the study group were identified among individuals with hearing loss who were routinely followed up in our clinic. Individuals with normal hearing who met the inclusion criteria were found among those who applied to the audiology clinic for various reasons (hoarseness, dizziness, etc.) for the control group. The study was explained to all participants and materials were sent to them via email between March and September 2021. Informed consent was obtained from all subjects and their caregivers.

### Materials

#### Social media attitude

The Social Media Attitude Scale was developed by Otrar and Argın to assess middle- and high-school students’ attitudes toward social media, considering both its advantages and disadvantages.^13^ The alpha value of the Social Media Attitude Scale was calculated as 0.85.

The scale comprises 23 items categorised into 4 subdimensions. The subdimension of social competence (six positive items) includes questions about the desire to express oneself, to be noticed and to gain prestige. The subdimension of the need for sharing (eight positive items) refers to sharing on social media sites, being aware of the shares and evaluating the claims. The subdimension of relationships with teachers (three positive items) assesses communication with teachers on social media and satisfaction. Finally, the social isolation subdimension (six negative items) addresses social media's impact on isolating individuals from their surroundings and diminishing their focus on lessons (reverse coding).

A high score indicates that students who answered the questions have positive attitudes toward social media. The scale's lowest score is 23 and its highest is 115.

#### Purposes of internet use

Akar developed the Internet Use Purposes Scale to assess adolescents’ internet usage motivations, encompassing communication, entertainment and information-seeking purposes. This validated scale comprises 5 subdimensions with a total of 29 items.^14^ Cronbach's alpha reliability coefficients for the scale's subdimensions range from 0.70 to 0.89. The overall Cronbach's alpha reliability coefficient is 0.86.

Within the education subdimension, questions pertain to activities such as homework and researching unfamiliar course topics. The entertainment sub-dimension addresses activities such as watching movies and serials, and playing video games. The psychological needs subdimension includes questions about alleviating loneliness and making new friends. The socialisation subdimension questions cover activities such as talking with friends and watching videos on video-sharing sites. Lastly, the information access subdimension encompasses activities such as staying updated on the news and learning new technologies.

The students were questioned about their agreement with the internet use purposes listed in the scale subdimensions. The scale responses range from 1 to 5, with 1 being ‘I strongly disagree’ and 5 being ‘I strongly agree.’ A high score indicates that the internet usage purpose in the subdimension is strong.

#### Problematic internet usage

Ceyhan *et al*. created the Problematic Internet Use Scale, for which the alpha value was calculated as 0.93.^15^ The Problematic Internet Use Scale is a validated scale that measures different facets of individuals’ internet usage behaviour and the corresponding psychological implications, including potential negative consequences, social benefits and excessive use tendencies.

Problematic Internet Use Scale includes 33 items and 3 subcategories. The negative consequences of internet use subscale evaluates the adverse outcomes and potential drawbacks arising from individuals’ internet use. It assesses behaviours and attitudes related to online interactions and their impact on individuals’ offline lives. The social benefit and/or social comfort subscale focuses on gauging the extent to which individuals turn to the internet for comfort and social support, particularly during times of emotional distress or when faced with personal problems. Finally, the excessive use subscale seeks to identify instances of internet overuse and their potential consequences on individuals’ daily lives. It examines respondents’ perceptions of disparities between their online and offline lives.

The scoring range for the scale is between 33 and 165, with higher scores indicative of potentially unhealthy internet usage, associated with adverse effects on one's life and an increased susceptibility to internet addiction.

#### Loneliness status

The University of California, Los Angeles Loneliness Scale is a validated instrument designed to measure feelings of social isolation and disconnection.^16^ The Turkish version of the scale, which underwent validity and reliability assessments conducted by Demir, was employed in this study.^17^ Its internal consistency (Cronbach's alpha) was found to be 0.96. The scale comprises 20 items, each describing situations reflecting thoughts and feelings concerning social relations. Respondents use a 4-point scale (1 = never, 4 = often) to report the frequency of their experiences with these situations. The scale consists of 10 positive items indicating the absence of semantic loneliness and 10 negative items suggesting the presence of semantic loneliness. The scale yields a maximum score of 80 and a minimum score of 20. A high score is regarded as indicating that loneliness is felt more intensely.

### Statistical analysis

The SPSS statistics 25.00 analysis program (IBM SPSS Inc., Armonk, NY, USA) was used for statistical evaluation. The data distribution was analysed using the Kolmogorov–Smirnov test, histograms and QQ plots. An independent samples *t*-test was used for between-group comparisons. Categorical variables were compared between groups using a chi-square test. Finally, the Pearson test was used to examine the relationship between the University of California, Los Angeles Loneliness Scale and the other questionnaires.

The statistical program G* Power 3.1 (Universitat Dusseldorf, Germany) was used to calculate the sample size.^18^ When the literature was examined, it was found that a significant difference between the internet usage habits of children with hearing loss and hearing peers has been reported, with an effect size of 1.0 (Cohen's *d*).^4^ It was therefore predicted that a similar level of difference would be detected between the problematic internet usage habits of the case and control groups in our study. It was calculated that 27 cases should be included in each group to detect this difference, with 95 per cent power and a 5 per cent type 1 error rate.

## Results

The demographics and clinical characteristics of the participants are shown in Table 1. The study and control groups had similar characteristics regarding the gender, age and educational status of subjects. In addition, there was no significant difference between the groups regarding daily internet usage time and internet usage days per week.

**Table 1.** Demographic information of adolescents in the groups

	Study group	Control group	Test statistic	*p*
Gender (*n* (%))				
– Female	14 (51.9)	18 (33.3)	1.227	0.268
– Male	13 (48.1)	9 (66.7)		
Age (years)	14.30 ± 1.81	14.63 ± 1.94	−0.651	0.518
Child's educational status (*n* (%))				
– Middle school	16 (59.3)	11 (40.7)	1.852	0.174
– High school	11 (40.7)	16 (59.3)		
Daily internet access (hours)	3.26 ± 1.97	2.85 ± 1.06	0.945	0.351
Weekly internet usage (days)	6.67 ± 0.73	6.78 ± 0.57	−0.618	0.539
Hearing device usage (*n* (%))				
– Unilateral cochlear implant	6 (22.2)			
– Bilateral cochlear implant	1 (3.7)			
– Bimodal	14 (51.9)			
Right ear hearing loss type (*n* (%))				
– Bilateral hearing aid	6 (22.2)			
– Sensorineural hearing loss	24 (88.9)			
– Conductive hearing loss	3 (11.1)			
Left ear hearing loss type (*n* (%))				
– Sensorineural hearing loss	24 (88.9)			
– Conductive hearing loss	3 (11.1)			
Right ear hearing loss degree (*n* (%))				
– Mild hearing loss	1 (3.7)			
– Moderate hearing loss	3 (11.1)			
– Severe hearing loss	10 (37.0)			
– Profound hearing loss	13 (48.1)			
Left ear hearing loss degree (*n* (%))				
– Moderate hearing loss	4 (14.8)			
– Severe hearing loss	14(51.9)			
– Profound hearing loss	9(33.3)			

A significant difference was found between the groups in the social isolation subscale and the Social Media Attitude Scale total score (*t* = −3.381, *p* = 0.001 and *t* = −2.022, *p* = 0.048, respectively). This difference was caused by individuals in the study group scoring lower than those in the control groups. There was no significant difference in the other Social Media Attitude Scale subscales.

While there was a statistically significant difference between the groups in terms of the education subscale of the Internet Use Purposes Scale (*t* = −2.087, *p* = 0.042), there was no significant difference in the other Internet Use Purposes Scale subscales. Individuals in the study group scored lower than those in the control group on the education subscale.

There was no significant difference between the groups in the Problematic Internet Use Scale social benefit and/or social comfort subscale. However, there were significant differences on the negative consequences subscale (*t* = 2.821, *p* = 0.007), the excessive use subscale (*t* = 2.382, *p* = 0.021) and the total score (*t* = 2.292, *p* = 0.005). This difference was caused by individuals in the study group scoring higher than those in the control groups.

There was no significant difference between the groups regarding the University of California, Los Angeles Loneliness Scale score (*t* = −0.525, *p* = 0.602). Table 2 displays the results of all comparisons.

**Table 2.** Comparison of variables between the groups

Scale	Study group (*n* = 27)	Control group (*n* = 27)	Test statistic	*p*
Social Media Attitude Scale (total)	72.78 ± 12.35	78.56 ± 8.22	−2.022	**0**.**048**
– Social competence	17.14 ± 5.27	16.74 ± 4.20	0.314	0.755
– Relationships with teachers	8.62 ± 3.48	7.77 ± 3.32	0.919	0.362
– The need for sharing	28.03 ± 5.93	30.48 ± 4.24	−1.741	0.88
– Social isolation	18.96 ± 5.55	23.55 ± 4.35	−3.381	**0**.**001**
Problematic Internet Use Scale (total)	90.93 ± 28.64	71.74 ± 17.022	2.292	**0**.**005**
– Excessive use	21.40 ± 4.94	18.48 ± 4.03	2.382	**0**.**021**
– Social benefit and/or social comfort	26.77 ± 9.6	22.33 ± 7.91	1.855	0.069
– negative consequences	15.66 ± 6.52	11.44 ± 4.23	2.821	**0**.**007**
Internet Use Purposes Scale				
– Education	24 ± 8.68	28.67 ± 7.73	−2.087	**0**.**042**
– Entertainment	20.48 ± 6.26	21.26 ± 4.99	−0.504	0.616
– Psychological needs	16.74 ± 5.15	15.33 ± 3.84	1.137	0.261
– Socialisation	15.56 ± 4.20	14.89 ± 3.08	0.664	0.51
– Information access	12.15 ± 3.48	12.93 ± 3.12	−0.864	0.391
University of California, Los Angeles Loneliness Scale	58.63 ± 7.85	59.89 ± 9.66	−0.525	0.602

In study group, there was a negative and moderate relationship between the University of California, Los Angeles Loneliness Scale and the social benefit and/or social comfort subscale and the total Problematic Internet Use Scale score (*r* = −0.369, *p* = 0.006 and *r* = −0.309, *p* = 0.023, respectively). On the other hand, a significant positive and moderate relationship was found between the University of California, Los Angeles Loneliness Scale and the socialisation subdimension of the Internet Use Purposes Scale (*r* = 0.360, *p* = 0.007).

## Discussion

This study compared the social media attitude, purpose of internet use, problematic internet usage and loneliness status of adolescents with hearing loss and with normal hearing.

According to our findings, adolescents with normal hearing have more positive attitudes toward social media and use the internet more for educational purposes than their hearing-impaired peers. The second significant finding was that teenagers with hearing impairment use the internet for entertainment, socialisation and psychological well-being, similar to their peers with normal hearing. However, research has also shown that adolescents with hearing loss have a higher risk of developing problematic internet usage and experiencing negative consequences. At the same time, those who feel more isolated tend to use the internet for socialising purposes.

### Social media attitude

Social engagement is a motivating strategy encouraging teenagers to actively use the internet.^19^ When we examined the teenagers’ attitudes toward social media, we found no significant differences between adolescents with normal hearing and those with hearing loss in terms of social competence, relationships with teachers and the need for sharing attitudes towards social media. Nevertheless, our study results indicate that adolescents with normal hearing experience less social isolation than their hearing-impaired peers, as reflected in their Social Media Attitude Scale scores. This result is consistent with the findings of Patel *et al*., who conducted a systematic review demonstrating that hearing impairment is associated with a higher prevalence of social isolation and loneliness.^20^

### Purpose of internet use

The internet has evolved into a versatile platform for those who are deaf or have hearing loss, providing a variety of opportunities ranging from educational opportunities to online support groups and sign language-based communication. While the internet has many benefits, it also has limitations, most notably the potential of encountering misinformation, which is a concern for this generation.^21^

The balance between educational and social pursuits is an important consideration in the context of internet usage among people with hearing loss. Disruptions in this balance can have a major influence on academic achievement and learning behavior.^22^ Our findings support this hypothesis, demonstrating that teenagers with hearing loss utilise the internet for entertainment, socialising, to access information and for psychological well-being at levels comparable to their peers. However, there is a distinct difference in educational internet use, with children with normal hearing making greater use of online resources for educational purposes. The difference may be linked to children with normal hearing having greater access to auditory information, which helps in their comprehension of online auditory resources.

The restricted use of the internet for educational reasons among people with hearing loss is an interesting finding in our study. The problem may be attributed in part to the absence of elements such as subtitles created specifically for this group in educational resources, a gap that remains in Turkey.^23^ Individuals with hearing loss can benefit greatly from the inclusion of online educational materials with captions or sign language interpretation, which can dramatically improve understanding of complicated topics and facilitate content consumption. In support of this notion, Kruger and Steyn highlighted the positive association between reading subtitles and academic achievement, implying that subtitles could give advantages in an academic setting.^24^ Furthermore, Chan *et al*.'s study emphasises the importance of the language used in subtitles, demonstrating that learners who read subtitles in their first language can improve their academic performance.^25^ As a result, providing accessible educational resources, such as subtitles and sign language interpretation, can be critical in fostering academic inclusion for those with hearing impairment.

With differences in internet usage purpose, people with hearing loss have a stronger tendency for personal and group contact online than their peers with normal hearing.^2^ Importantly, our study found no significant differences in daily internet usage time and frequency of internet use per week between the hearing loss group and their peers with normal hearing. These findings are consistent with recent research by Thorén *et al*., who found that individuals with hearing loss and their age-matched counterparts with normal hearing use computers and the internet at comparable rates.^26^ This consistency in findings highlights the potential of technology as an effective tool for bridging the knowledge and communication gap between those with hearing loss and the general population.

### Problematic internet usage

The increase in internet use and the advancement of digital communication platforms have resulted in problematic internet use, which is characterised by individuals’ inability to control their online activities, causing distress and interfering with daily functioning.^27,28^ Wu *et al*. conducted a study investigating the prevalence of internet addiction and its association with social support and other related factors among adolescents in China. The findings revealed that the prevalence of internet addiction among adolescents was 10.40 per cent, with boys exhibiting a higher susceptibility than girls. Several significant factors were identified as contributors to internet addiction, including poor self-control, low self-esteem, feelings of loneliness, a lack of parental care and the pressures of academic demands. Additionally, the study emphasised the protective impact of social support, emphasising its ability to reduce the risk of internet addiction.^29^

Michalczyk's study extended the investigation of problematic internet use to those with hearing loss, revealing a higher tendency for problematic internet use, a pervasive sense of loneliness and negative emotional experiences among the participants.^30^

In line with previous research, our study found that adolescents with hearing loss had higher levels of excessive internet use, negative consequences and total Problematic Internet Use Scale score than their counterparts with normal hearing. Notably, there was no significant difference between the two groups on the social benefit or comfort subscale. Excessive internet use resulted in increased social isolation and negative consequences, emphasising the significance of promoting balanced and appropriate internet and social media use among teenagers. This highlights the importance of advice and support, especially for children with hearing impairments, in ensuring healthy and productive involvement with digital platforms.

Our findings emphasise hearing-impaired adolescents’ risk of problematic internet use, emphasising the importance of designing educational and intervention programmes specific to this community. Adolescents with hearing loss who are struggling with internet addiction need the support and encouragement of their parents, teachers and classmates. A previous study showed that social support is a protective factor against problematic internet use.^29^

Further research in this area is required to clarify the complex aspects that contribute to problematic internet use, particularly among adolescents with hearing loss. Insights obtained from such investigations can be used to develop specific interventions to address this developing challenge.

### Loneliness

Our findings showed that there was no significant difference in feelings of loneliness between adolescents with hearing loss and those with normal hearing. This finding contrasts with the findings of Majorano *et al*., who found that teenagers with cochlear implants experienced higher degrees of loneliness than their hearing peers.^31^ A scoping study of social isolation and loneliness among hearing-impaired children and adolescents also showed a possible relationship between hearing loss and social isolation, with negative consequences for overall well-being.^20^

Conversely, Adigun *et al*. presented a different perspective, demonstrating no significant relationship between social media use and loneliness in hearing-impaired students.^32^ On a similar subject, Barak and Sadovsky reported surprising findings demonstrating that deaf participants with reduced internet use had higher feelings of loneliness and poorer self-esteem than their hearing counterparts. Those who used the internet more frequently, on the other hand, had higher overall well-being.^4^

Our study found that there was a negative relationship between the University of California, Los Angeles Loneliness Scale and the social benefit and/or social comfort subscale and the total score of the Problematic Internet Use Scale. This shows that the individual's tendency for internet addiction was connected to lower feelings of loneliness. Furthermore, people who used the internet to get comfort and social support had decreased levels of loneliness. In addition, there was a moderate and positive correlation between the socialisation subdimension of the Internet Use Purposes Scale and the University of California, Los Angeles Loneliness Scale loneliness score. This suggests that the internet and social media can be important tools for social connection and communication for children, including those with hearing impairment.

Individuals who experience higher levels of loneliness tended to use the internet more frequently for socialising purposes, such as chatting with friends and watching videos on video-sharing websites. Based on this data, we can conclude that internet use among our participants reduced feelings of loneliness. However, it is important to note that while the internet may temporarily relieve loneliness, excessive internet use can worsen feelings of isolation and lead to negative consequences, such as social withdrawal and reduced face-to-face interactions.

- Adolescents with hearing loss are known to utilise the internet more extensively than their hearing counterparts, particularly for personal and group communication
- Adolescents with normal hearing have more access to educational resources online, while those with hearing loss may face limitations
- Teenagers with hearing impairment use the internet for entertainment, socialisation and psychological well-being, similar to their peers with normal hearing
- Adolescents with hearing loss have a higher risk of developing problematic internet usage and experiencing negative consequences
- Persons who used the internet for social support and comfort reported lower degrees of loneliness

There are many ways for adolescents with hearing loss to relieve loneliness and build social connections that do not involve spending much time on the internet. They can improve their overall well-being and quality of life by seeking social support, engaging in physical activity, learning new skills and seeking professional help.

### Strengths and limitations

The present study has several strengths, such as the inclusion of the patients using a hearing aid and cochlear implant, and the exclusion of the subjects not using amplification devices. However, the study also has several limitations. The major limitation is only using the questionnaires and performing them via the online platform. In addition, our analysis was limited to the data documented in patient self-reported information. Another limitation of the study is the absence of consideration of the age at which hearing loss began and the duration of the hearing loss. Future research should concentrate determining the age at which hearing loss begins. This will allow us to more precisely assess the impact of hearing loss duration on internet usage.

## Conclusion

This study highlights significant differences in internet use patterns between adolescents with normal hearing and those with hearing impairment. We found that children with normal hearing have more access to educational resources online, while those with hearing loss may face limitations. Additionally, our research emphasises the dangers of problematic internet use among hearing-impaired adolescents, underlining the significance of educational activities and social assistance. Furthermore, while internet use can help to reduce loneliness among all participants, excessive use can have negative consequences. Exploring alternative ways for adolescents with hearing loss to build social connections and address feelings of loneliness is therefore important.

Our findings have implications for educators and mental health professionals working with adolescents with hearing impairment and suggest a more inclusive educational and social media resource design. Further research is needed to understand better the complex relationship between internet use, loneliness and well-being in this population.

## Data Availability

The data that support the findings of this study are available from the corresponding author upon reasonable request.
